# Comparative analysis of flavonoid metabolites from different parts of *Hemerocallis citrina*

**DOI:** 10.1186/s12870-023-04510-6

**Published:** 2023-10-13

**Authors:** Hongrui Lv, Shang Guo

**Affiliations:** Shanxi Institute for Functional Food, Shanxi Agricultural University, No.79, Longcheng Street, Taiyuan City, Shanxi Province China

**Keywords:** *Hemerocallis citrina*, Widely targeted metabolomics, Flavonoid metabolites

## Abstract

**Background:**

*Hemerocallis citrina* Baroni is a traditional medical and edible plant. It is rich in flavonoid compounds, which are a kind of important bioactive components with various health benefits and pharmaceutical value. However, the flavonoid metabolomics profile and the comparison of flavonoid compounds from different parts of *H. citrina* is scarce.

**Results:**

In this study, flavonoid metabolites were investigated from roots, stems, leaves and flowers of *H. citrina*. A total of 364 flavonoid metabolites were identified by UPLC-MS/MS based widely targeted metabolomics, and the four plant parts showed huge differences at flavonoid metabolic level. Compared to roots, 185, 234, and 119 metabolites accounted for upregulated differential flavonoid metabolites (DFMs) in stems, leaves, and flowers, respectively. Compared to stems, 168 and 29 flavonoid metabolites accounted for upregulated DFMs in leaves and flowers, respectively. Compared to leaves, only 29 flavonoid metabolites accounted for upregulated DFMs in flowers. A number of 35 common flavonoid metabolites were observed among six comparison groups, and each comparison group had its unique differential metabolites. The most abundant flavonoid metabolites in the four parts are flavonols and flavones, followed by flavanones, chalcones, flavanols, flavanonols, anthocyanidins, tannin, and proanthocyanidins. 6,7,8-Tetrahydroxy-5-methoxyflavone, 7,8,3’,4’-tetrahydroxyflavone, 1-Hydroxy-2,3,8-trimethoxyxanthone, Farrerol-7-*O*-glucoside, 3’,7-dihydroxy-4’-methoxyflavone, 3,3’-*O*-Dimethylellagic Acid, 5-Hydroxy-6,7-dimethoxyflavone, Nepetin (5,7,3’,4’-Tetrahydroxy-6-methoxyflavone), (2s)-4,8,10-trihydroxy-2-methoxy-1 h,2 h-furo[3,2-a]xanthen-11-one are dominant in roots. Isorhamnetin-3-*O*-(6’’-malonyl)glucoside-7-*O*-rhamnoside, 7-Benzyloxy-5-hydroxy-3’,4’-methylenedioxyflavonoid, 3-Hydroxyphloretin-4’-*O*-glucoside are dominant in stems. Chrysoeriol-7-*O*-glucoside, Epicatechin glucoside, Kaempferol-3-*O*-rhamnoside (Afzelin)(Kaempferin)*, Azaleatin (5-*O*-Methylquercetin), Chrysoeriol-5-*O*-glucoside, Nepetin-7-*O*-glucoside(Nepitrin), 3,5,7,2’-Tetrahydroxyflavone; Datiscetin, Procyanidin B2*, Procyanidin B3*, Procyanidin B1, Isorhamnetin-3-*O*-(6’’-acetylglucoside) are dominant in leaves. kaempferol-3-p-coumaroyldiglucoside, Delphinidin-3-*O*-sophoroside-5-*O*-glucoside, Limocitrin-3-*O*-sophoroside, Kaempferol-3-*O*-rutinoside(Nicotiflorin), Luteolin-7-*O*-(6’’-malonyl)glucoside-5-*O*-rhamnoside are dominant in flowers.

**Conclusion:**

There was significant difference in flavonoid metabolites among different parts of *H. citrina*. Leaves had relative higher metabolites contents than other parts. This study provided biological and chemical evidence for the different uses of various plant parts of *H. citrina*, and these informations are important theoretical basis for the food industry, and medical treatment.

**Supplementary Information:**

The online version contains supplementary material available at 10.1186/s12870-023-04510-6.

## Background

*Hemerocallis citrina* Baroni is a species of perennial herb belonging to the family of Asphodelaceae [1]. *H. citrina* has been widely grown in China, Japan, and Korea, and its flower buds are a kind of famous vegetable widely consumed in Asia [2, 3]. Datong city of Shanxi province is one of the main *H. citrina* producing areas in China, where the land is rich in selenium, the diurnal temperature variation is 20 ℃, and air and water are not polluted [4]. These advantageous phenological conditions make flower buds a real selenium rich Green food with the characteristics of larger horn and thicker flesh, more brittle and tender, more golden color than *H. citrina* from other regions, which appeal to domestic and foreign consumers [5]. *H. citrina* is also a traditional medicinal plant, which has been written in ancient books for thousands of years [6]. According to a famous Chinese encyclopedia of medicine “Compendium of Materia Medica” in the Ming dynasty, the flower buds of *H. citrina* have been recorded to alleviate depression [3, 7]. Modern pharmacological studies indicated that *H. citrina* contains not only proteins, minerals and other nutrients, but also polysaccharides, phenols, flavonoids, anthraquinones and other bioactive compounds. Flavonoid compounds from *H. citrina* is a research focus of natural pharmaceutical chemistry as for its crucial bioactivities of anti-depression, antioxidant, anti-inflammatory, anti-cancer and so on [8].

In plants, the distribution of secondary metabolites is generally specific to species, organs, tissues, and growth periods [9]. In different parts of the same plant species, there are usually wide differences with respect to the types and quantities of metabolites [10]. For example, the antioxidant activity of *Rumex tunetanus* flowers was significantly higher than its stems due to the chemical components differences between distinct plant parts [11]. To our knowledge, most of the previous researches carried out on *H. citrina* were restricted to its flower buds. Consequently, there is a relatively better comprehension of the flower buds part. However, the metabolites variety of other plant parts such as roots, stems and leaves in *H. citrina* have not been well studied.

Technical development in compounds large-scale identification have enabled analysis of metabolites varieties in plants. The researches utilizing metabolomics is able to identify and profile the bioactive compounds with health benefits, which can exploit its new usages [12]. The widely targeted metabolomics based metabolites analysis is a crucial method to have a whole view of the dominant flavonoid compounds exist in different parts of the plant. This study aimed to compare the flavonoid metabolites based on widely targeted metabolomics of different plant parts of *H. citrina*. This study provided the foundation for further investigation for the different usages of various plant parts of *H. citrina*, and these informations are important scientific basis for the food industry, and medical treatment.

## Results

### Mass spectrum data evaluation

**Fig. 1:**
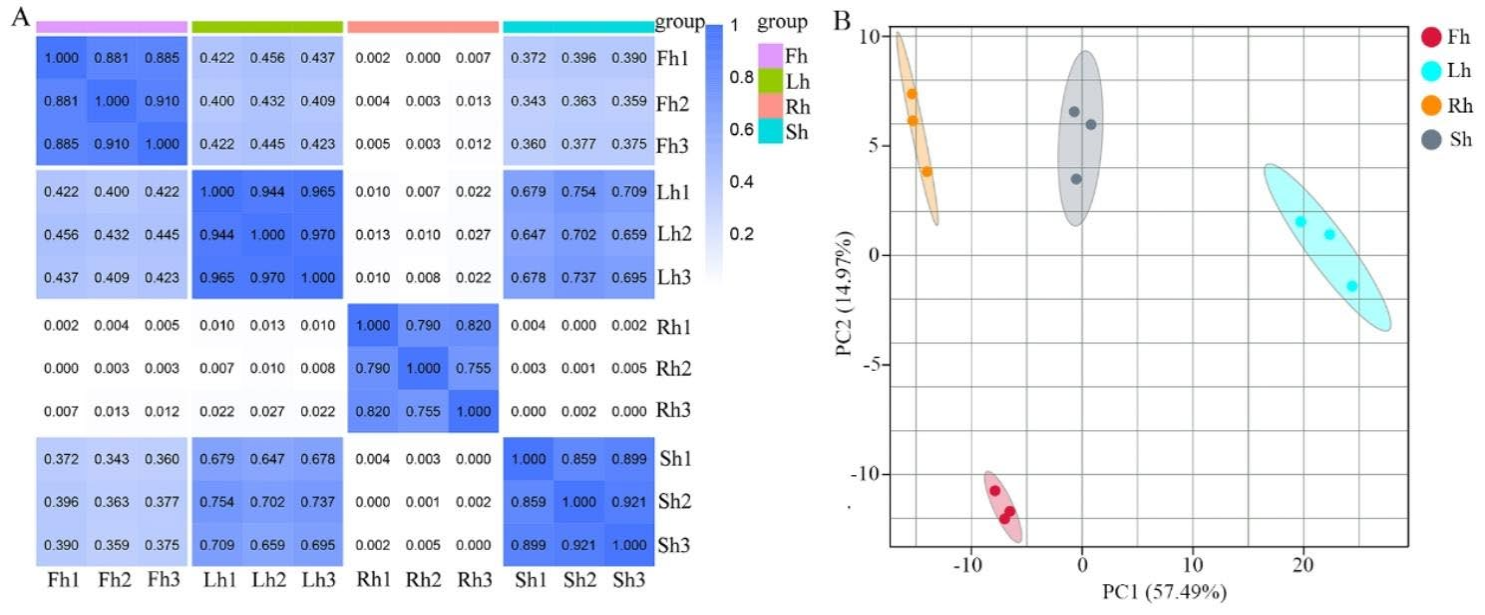
Spearman correlation analysis and principal component analysis of 12 samples from *H. citrina* roots, stems, leaves, and flowers. **(A)** Spearman correlation analysis. The darker the blue color in the correlation analysis heatmap, the higher the degree of correlation between samples. The correlation coefficient between each triplicate measurements was greater than 0.7; **(B)** Principal component analysis. The small symbols with different colors represent different groups of samples. The percentages of the explained values of PC1 and PC2 were 57.49% and 14.97%, respectively

To obtain reliable and high-quality *H. citrina* flavonoid metabolome data, spearman correlation analysis (SCA) and principal component analysis (PCA) were performed on 12 samples from its roots (Rh), stems (Sh), leaves (Lh), and flowers (Fh) in this study. The SCA coefficient between the three replicated samples was greater than 0.7, which indicated that the experiment was repeatable and reliable for the Rh, Sh, Lh and Fh groups (Fig. 1A). PCA is a tool that uses a small number of principal components to reveal the internal structure among multiple variables. The PCA results showed that the percentages of the explained values of PC1 and PC2 were 57.49% and 14.97%, respectively (Fig. 1B). The PCA clearly separated the plant parts into four clusters, suggesting huge differences among the roots, stems, leaves, and flowers at the flavonoid metabolic level.

### Overview of the differential flavonoid metabolites (DFMs)

**Fig. 2:**
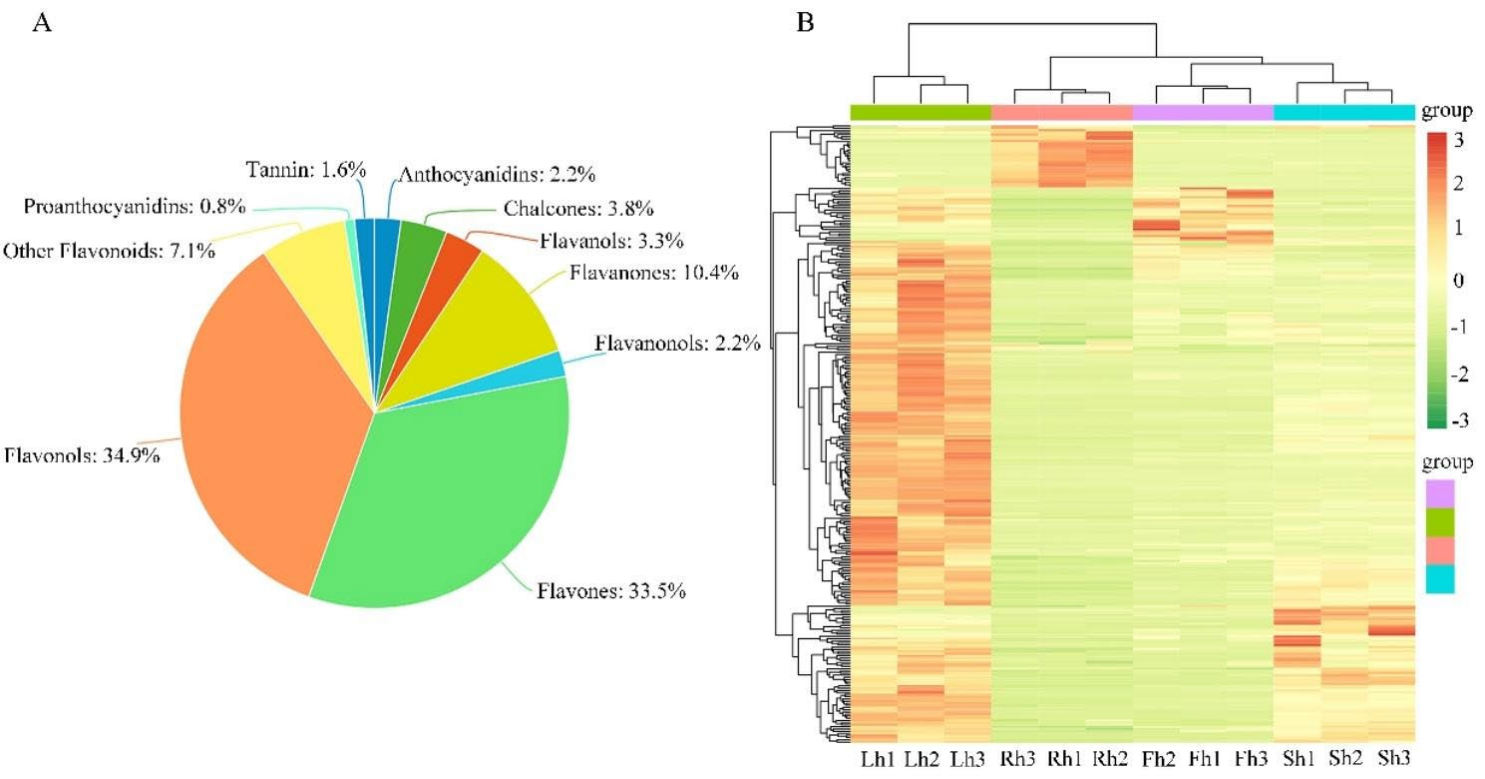
The variations in the abundance of DFMs among the four *H. citrina* parts based on metabolomic profiling. **(A)** Pie chat of the numbers of DFMs belonging to major metabolic categories; **(B)** A heatmap of the abundance of DFMs of the four selected parts. The sample name is horizontal, Rt1-3, St1-3, Lt1-3, Ft1-3 were the samples of roots, stems, leaves, flowers, respectively. The metabolite information is vertical, and the different colors are the values after the relative content standardization (red represents high content, green represents low content)

To better understand the differences in flavonoid metabolites among the different parts of *H. citrina*, ultra performance liquid chromatography–tandem mass spectrometry (UPLC-MS/MS) based widely targeted metabolomics of roots, stems, leaves, and flowers was performed. A total of 364 metabolites of flavonoid compounds were identified from these four plant parts (Table S1). These DFMs consist of flavonols (34.9%), flavones (33.5%), flavanones (10.4%), chalcones (3.8%), flavanols (3.3%), flavanonols (2.2%), anthocyanidins (2.2%), tannin (1.6%), proanthocyanidins (0.8%), and other flavonoids (7.1%) (Fig. 2A). All detected flavonoid metabolites were shown in a heatmap after homogenization. The metabolite profiling of the Rh, Sh, Lh, and Fh groups showed huge variations in their flavonoid metabolomes (Fig. 2B), which indicated that there were significant differences in the metabolite levels of the four plant parts. By clustering all flavonoid metabolites, it was revealed that more than half of the flavonoid metabolites in leaves were present at higher levels than roots, stems, and flowers.

### DFMs screening, functional annotation, and enrichment analysis

**Fig. 3:**
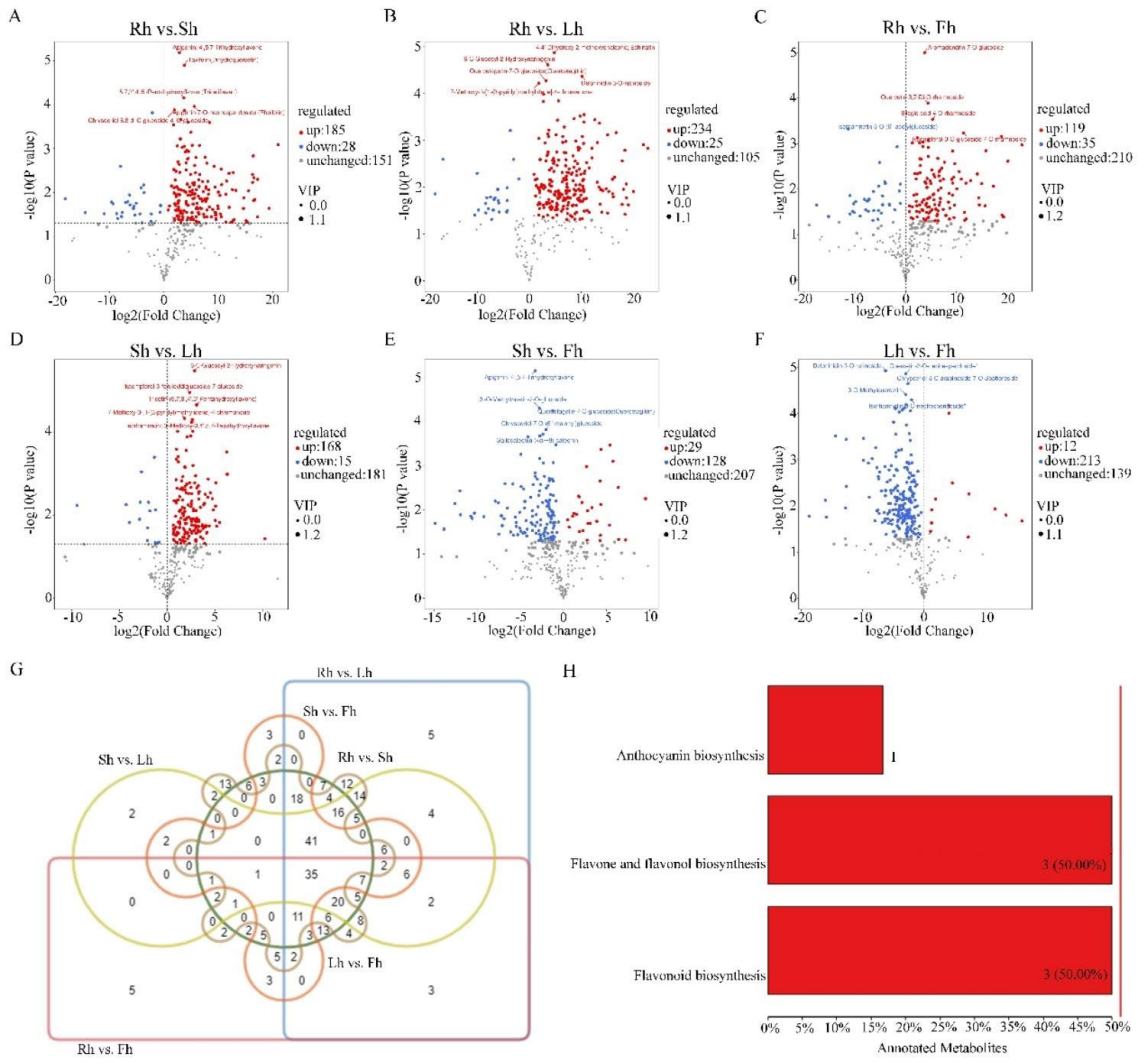
DFMs screening and enrichment analysis. **(A-F)** The volcano plots of DFMs in the group of Rh vs. Sh, Rh vs. Lh, Rh vs. Fh, Sh vs. Lh, Sh vs. Fh, Lh vs. Fh, respectively. The threshold P < 0.05 was used to determine the significance of DFMs. Red and blue dots represent upregulated and downregulated flavonoid metabolites, respectively, and gray dots indicate DFMs that did not change significantly. The size of the dot represents the VIP value. The larger the dot, the larger the VIP value, and the more reliable the differentially expressed metabolites obtained. Top five metabolites were identified according to P value ranking and marked in the figures; **(G)** Venn diagram of DFMs between different comparison groups; **(H)** KEGG pathway enrichment of DFMs. Copyright permission was granted for using and adapting the KEGG image by Copyright holder, the Kanehisa Laboratories [13]

To gain more insight into the differential flavonoid metabolites between the four *H. citrina* parts, DFMs were filtered according to the criterion of fold change (FC) > 1, variable importance in the projection (VIP) ≥ 1 and P < 0.05. The filtering results were illustrated by volcano plots and venn diagram (Fig. 3). Compared to *H. citrina* roots, 213, 259, and 154 flavonoid metabolites were differentially expressed in stems, leaves, and flowers respectively, and among them, 185, 234, and 119 metabolites were accounted for upregulated DFMs (Fig. 3A-C). Compared to stems, 183, and 157 flavonoid metabolites were differentially expressed in leaves and flowers, respectively, and among them, 168 and 29 metabolites were accounted for upregulated DFMs (Fig. 3D-E). There are 154 DFMs in the group of Lh vs. Fh, including 12 upregulated flavonoid metabolites and 213 downregulated flavonoid metabolites (Fig. 3F). Most of the flavonoid metabolites of roots, stems and flowers were downregulated relative to leaves, which indicated that there were more flavonoid metabolites in leaves than in the other three parts.

After taking intersection of each comparison group in a venn diagram (Fig. 3G), 35 common DFMs were observed among six comparison groups. Each comparison group had its unique differential metabolites. Therefore, differential metabolites could clearly distinguish roots, stems, leaves, and flowers from each other. The annotated *H. citrina* flavonoid metabolites were mapped to the kyoto encyclopedia of genes and genomes (KEGG) pathways to investigate the DFMs involved in important metabolic pathways (Fig. 3H). The results showed that epigallocatechin, quercetin and catechin were involved in flavonoid biosynthesis pathway. Peonidin-3-*O*-glucoside was enriched in anthocyanin biosynthesis pathway. Quercetin, acacetin, and 3,7-Di-*O*-methylquercetin participated in flavone and flavonol biosynthesis pathway (Table S2).

### Identification of the dominant flavonoid metabolites in different plant parts

**Fig. 4:**
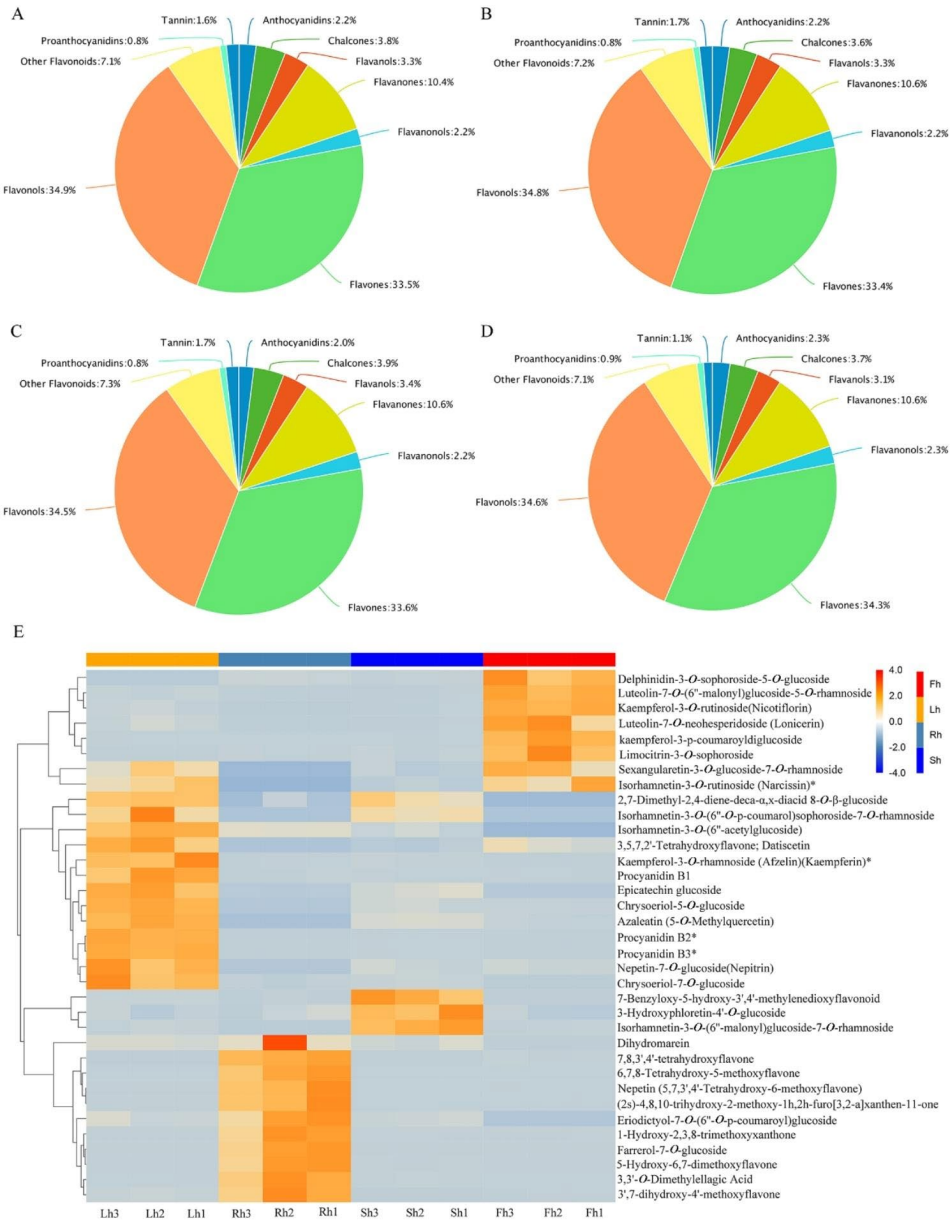
Dominant flavonoid metabolites screening in different plant parts. **(A-D)** Pie charts of the proportions of flavonoid metabolites belonging to major categories in roots, stems, leaves, flowers, respectively; **(E)** Heatmap of dominant flavonoid metabolites in different plant parts. The sample name is horizontal, Rt1-3, St1-3, Lt1-3, Ft1-3 were the samples of roots, stems, leaves, flowers, respectively. The metabolite information is vertical, and the different colors are the values after the relative content standardization (red represents high content, blue represents low content)

Of the 364 flavonoid metabolites, a total of 323 DFMs were identified in roots. A total of 359 DFMs were identified in stems. A total of 357 DFMs were identified in leaves. A total of 350 DFMs were identified in flowers. As shown in Fig. 4A-D, the most abundant flavonoid metabolites in roots, stems, leaves, and flowers are flavonols and flavones, followed by flavanones, chalcones, flavanols, flavanonols, anthocyanidins, tannin, and proanthocyanidins.

Figure 4E is the screening results of dominant flavonoid metabolites in different plant parts of root, stem, leaf and flower, based on FC > 1, VIP ≥ 1, P < 0.05. A total of 35 DFMs were analyzed. 6,7,8-Tetrahydroxy-5-methoxyflavone, 7,8,3’,4’-tetrahydroxyflavone, 1-Hydroxy-2,3,8-trimethoxyxanthone, Farrerol-7-*O*-glucoside, 3’,7-dihydroxy-4’-methoxyflavone, 3,3’-*O*-Dimethylellagic Acid, 5-Hydroxy-6,7-dimethoxyflavone, Nepetin (5,7,3’,4’-Tetrahydroxy-6-methoxyflavone), (2s)-4,8,10-trihydroxy-2-methoxy-1 h,2 h-furo[3,2-a]xanthen-11-one, presented relative higher contents in roots than stems, leaves, and flowers. Isorhamnetin-3-*O*-(6’’-malonyl)glucoside-7-*O*-rhamnoside, 7-Benzyloxy-5-hydroxy-3’,4’-methylenedioxyflavonoid, 3-Hydroxyphloretin-4’-*O*-glucoside, presented relative higher contents in stems than roots, leaves, and flowers. Chrysoeriol-7-*O*-glucoside, Epicatechin glucoside, Kaempferol-3-*O*-rhamnoside (Afzelin)(Kaempferin)*, Azaleatin (5-*O*-Methylquercetin), Chrysoeriol-5-*O*-glucoside, Nepetin-7-*O*-glucoside(Nepitrin), 3,5,7,2’-Tetrahydroxyflavone; Datiscetin, Procyanidin B2*, Procyanidin B3*, Procyanidin B1, Isorhamnetin-3-*O*-(6’’-acetylglucoside), presented relative higher contents in leaves than roots, stems, and flowers. kaempferol-3-p-coumaroyldiglucoside, Delphinidin-3-*O*-sophoroside-5-*O*-glucoside, Limocitrin-3-*O*-sophoroside, Kaempferol-3-*O*-rutinoside(Nicotiflorin), Luteolin-7-*O*-(6’’-malonyl)glucoside-5-*O*-rhamnoside, presented relative higher contents in flowers than roots, stems, and leaves. In addition, the relationships between the compounds that are predominate in roots, stems, leaves, and flowers, respectively, and the hundreds of others is really complicated (Fig. S1). 6,7,8-Tetrahydroxy-5-methoxyflavone, 7,8,3’,4’-tetrahydroxyflavone, 1-Hydroxy-2,3,8-trimethoxyxanthone, which are dominant in roots, presented highly negative correlation with most other differential metabolites. The dominant flavonoid metabolites in leaves showed significantly positive correlation with most other flavonoid metabolites. The correlation between the predominate flavonoid metabolites in stems and flowers and most other flavonoid metabolites was found to be not significant.

## Discussion

*H. citrina*, namely “Huang Hua Cai” in Chinese, has been widely cultivated in Asia, which is a medicinal and edible homologous vegetable, and its flower buds are one of the popular consumed foods [14]. Traditionally, the roots, leaves, and flowers of *Hemerocallis* plants have been used as medicine for thousands of years [15]. The flowers of *H. citrina* were used for alleviating swelling, haemostasis, jaundice, treating breast carbuncle, and improving sleep, and the roots of *Hemerocallis thunbergii* were used for the therapy of schistosomiasis, which have been recorded in the famous ancient medical book ‘Compendium of Materia Medica’ [16]. The leaves from *Hemerocallis fulva* are used for relieving inflammation, jaundice, and insomnia [17]. Modern pharmacological studies indicated that flavonoid compounds are one of the important bioactive components in *H. citrina*, and have lots of beneficial functions, such as preventing and treating cardiovascular and cerebrovascular diseases, alleviating cough, scavenging bacteria, protecting liver, reducing and inhibiting free radicals as antioxidants [18]. The study indicated that flavonoids widely exist in roots, stems, leaves, and flowers of *H. citrina*.

The relative metabolite levels in different plant parts represent the holistic nutritions and phytochemicals features and distribution [19], which assist in discovering the most advantageous plant parts for further targeted bioactive metabolites study. Previously, only a total of 41 flavonoid compounds were found in *Hemerocallis* species, including 33 compounds identified from flowers, 5 compounds from leaves, and 3 compounds from roots [4]. In the present study, the widely targeted metabonomics analysis of the extracts from roots, stems, leaves, and flowers of *H. citrina* indicated 364 metabolites belonging to flavonoid compounds. The repeatability of the three biological samples of each group was well, and the flavonoid metabolites accumulating in roots, stems, leaves, and flowers were clustered into four distinct clusters, respectively. The flavonoid metabolites involved in roots were less abundant than in other parts, indicating that these absent metabolites could be particular to the aboveground parts of *H. citrina*. In another study with respect to rice, the accumulation of most flavonoid metabolites in roots was the lowest among different tissues [10], which has the similar results with the present study.

Based on the P value, the top five DFMs in the comparison of Rh vs. Sh were Apigenin; 4’,5,7-Trihydroxyflavone, Taxifolin (Dihydroquercetin), 5,7,3’,4’,5’-Pentahydroxyflavan (Tricetiflavan), Apigenin-7-*O*-neohesperidoside (Rhoifolin), Chrysoeriol-6,8-di-C-glucoside-4’-*O*-glucoside (Fig. 3A). Taxifolin shows antioxidant activity and radical scavenging activity [20, 21]. Tricetiflavan possesses antiviral activity [22]. Apigenin-7-neohesperidoside (Rhoifolin) in carrageenin-induced rat edema model present anti-inflammatory effect [23]. The top five DFMs in the comparison of Rh vs. Lh were 4,4’-Dihydroxy-2-methoxychalcone; Echinatin, 6-C-Glucosyl-2-Hydroxynaringenin, Delphinidin-3-*O*-rutinoside, Quercetagetin-7-*O*-glucoside(Quercetagitrin), 7-Methoxy-3-[1-(3-pyridyl)methylidene]-4-chromanone (Fig. 3B). Echinatin has great advantages of hepatoprotective antioxidant and anti-inflammatory effect [24]. Quercetagetin-7-*O*-glucoside shows pivotal antioxidant ability to scavenge free radicals [25]. In the comparison of Rh vs. Fh, Aromadendrin-7-*O*-glucoside, Quercetin-3,7-Di-*O*-rhamnoside, Ellagic acid-4-*O*-rhamnoside, Isorhamnetin-3-*O*-(6’’-acetylglucoside), Kaempferol-3-*O*-glucoside-7-*O*-rhamnoside were the top five DFMs (Fig. 3C). However, the function of these compounds is scarce. 6-C-Glucosyl-2-Hydroxynaringenin, kaempferol-3-feruloyldiglucoside-7-glucoside, Tricetin (5,7,3’,4’,5’-Pentahydroxyflavone), 7-Methoxy-3-[1-(3-pyridyl)methylidene]-4-chromanone, Isorhamnetin; 3’-Methoxy-3,4’,5,7-Tetrahydroxyflavone were the top five DFMs in the comparison of Sh vs. Lh (Fig. 3D). Tricetin is a dietary flavonoid and could restrain the proliferation and migration of various cancer cells [26–28]. Isorhamnetin also decreases skin cancer and gastric cancer [29, 30]. The top five DFMs were Apigenin; 4’,5,7-Trihydroxyflavone, 3’-*O*-Methyltricetin-7-*O*-glucoside, Quercetagetin-7-*O*-glucoside(Quercetagitrin), Chrysoeriol-7-*O*-(6’’-malonyl)glucoside, Gallocatechin-(4α→8)-catechin in the comparison of Sh vs. Fh (Fig. 3E). Apigenin reduces lipid peroxidation in mice [31] and suppresses growth and development of human anaplastic thyroid cancer cells [32]. In the formalin test, Gallocatechin-(4α→8)-catechin administered orally showed an antinociceptive effect [33]. The top five DFMs were Delphinidin-3-*O*-rutinoside, Quercetin-3-*O*-neohesperidoside*, Chrysoeriol-8-C-arabinoside-7-*O*-Sophoroside, 3-*O*-Methylquercetin, and Isorhamnetin-3-*O*-neohesperidoside* in the comparison of Lh vs. Fh (Fig. 3F). Delphinidin-3-*O*-rutinoside related to regulating the redox-sensitive caspase 3-related pro-apoptotic effect of blackcurrant juice on leukaemia Jurkat cells [34]. Quercetin 3-*O*-neohesperidoside, a flavonoid glycoside, has antimicrobial activity [35]. In vitro studies indicated that 3-*O*-Methylquercetin protected major cereals from phytopathogenic fungi [36]. Isorhamnetin 3-*O*-neohesperidoside from *Acacia salicina* has protective effects toward oxidation damage and genotoxicity induced by aflatoxin B1 and nifuroxazide [37]. There were 35 common DFMs in the six comparison groups (Fig. 3G), which revealed that these common DFMs may act as a pivotal and indispensable role in various plant parts.

Based on the quantitative analysis of the detected DFMs, the dominant flavonoid metabolites in *H. citrina* roots, stems, leaves, and flowers were screening. As for the dominant flavonoid metabolites in roots, 7,8,3’,4’-tetrahydroxyflavone is specific inhibitors of vertebrate Inositol-1,4,5-trisphosphate 3-Kinases C [38], and has the antitrypanosomal and antileishmanial activities [39]. 3’,7-dihydroxy-4’-methoxyflavone was firstly discovered in seeds of *Acacia farnesiana* [40]. The dominant flavonoid metabolites in stems were rarely reported. As for the dominant flavonoid metabolites in leaves, Chrysoeriol-7-*O*-glucoside was found mostly in plant leaves, and also determined the yellow color as copigments [41]. Chrysoeriol-7-*O*-glucoside exhibits antiviral and antioxidant effects [42]. Epicatechin glucoside in broad bean seed extract showed antioxidant and radical-scavenging properties [43]. Kaempferol-3-*O*-rhamnoside presents inhibitory effects on inflammation [44], and suppresses MCF-7 breast cancer cell proliferation by activation of the caspase cascade pathway [45]. 3,5,7,2’-Tetrahydroxyflavone shows a strong fungitoxic activity towards *Fusarium oxysporum* [46]. Procyanidin B2 protects lipopolysaccharide-induced myocardial from cell apoptosis [47] and has protective effect on acute liver damage [48]. Procyanidin B1 presents anti-inflammatory [49], antioxidant activity and anti-apoptotic effect [50]. As for the dominant flavonoid metabolites in flowers, Kaempferol-3-*O*-rutinoside has antiglycation [51], anti-hypertensive [52], and anti-cancer activity [53]. The distribution of dominant metabolites in different parts has complicated characteristics, indicating that the chemical or pharmacological components of plant parts have the potential for diversity [54]. Of the 364 metabolites, the number of 41, 5, 7, 14 flavonoid compounds were respectively missing in roots, stems, leaves, and flowers, which demand for increase our knowledge of the functional relevance between the metabolites and the plant parts.

## Conclusions

In the present study, the composition characteristics of flavonoid metabolites in different plant parts of *H. citrina* were assessed by widely targeted metabolomics technology. These informations are of value for the food industry, medical treatment, and health benefits. A total of 364 metabolites of flavonoid compounds were identified from roots, stems, leaves, and flowers. The most abundant flavonoid metabolites were flavonols and flavones, followed by flavanones, chalcones, flavanols, flavanonols, anthocyanidins, tannin, and proanthocyanidins. There were significant differences at flavonoid metabolite levels of the four plant parts. More than half of the flavonoid metabolites in leaves were present at higher levels than roots, stems, and flowers. And the metabolites distributed in roots were less abundant than in other parts. To our knowledge, this work marks the first systematical comprehensive of flavonoid metabolites from different parts of *H. citrina*, and provided the scientific basis for further investigation for the different usages of various plant parts.

## Materials and methods

### Plant material and preparation of extracts

The materials *Hemerocallis citrina* cv. ‘Datonghuanghua’ were collected from Yuzhou district of Datong city, Shanxi province, China (40°1′ N, 113°39′ E, altitude 1042 m) in August, 2022, and were unambiguously identified by Doctor Shang Guo (Shanxi Agricultural University). The voucher specimen is stored in the Institute for Functional Food, Shanxi Agricultural University, with the deposition number HuangHuaCai-20220815. The plants growing conditions such as light intensity, air temperature and humidity were depending on outside environment. Fifteen uniformly healthy *H. citrina* plants were pooled as a single biological replicate and separated into four parts (root, stem, leaf and flower), immediately frozen with liquid nitrogen and then transferred to -80 ℃ for preservation. When prepared to extract, samples were placed in a lyophilizer (Scientz-100 F, SCIENTZ, Ningbo, China) for vacuum freeze-drying. Then, the samples were ground to powder with a grinder (MM 400, Retsch, Haan Germany), which was operated at 30 Hz, 1.5 min. 100 mg of powder was weighed and metabolites were extracted with 1.2 mL of 70% methanol, followed by vortex once every 30 min, a total of 6 times, and each time lasting for 30 s [55]. Each plant part sample had three independent replicates for metabolites extraction. The samples were placed in the refrigerator at 4 ℃ overnight. The mixtures were centrifuged at 12,000 rpm for 3 min. Then, the supernatant was filtered by microfiltration membrane (0.22 μm pore size) before UPLC-MS/MS analysis.

### UPLC conditions

The extracts were analysed utilizing an ultra-performance liquid chromatography-electrospray ionization-tandem mass spectroscopy (UPLC-ESI-MS/MS) system (UPLC, ExionLC™ AD, https://sciex.com.cn/; MS/MS, Applied Biosystems 6500 QTRAP, https://sciex.com.cn/). The analysis conditions were as follows, chromatographic column, Agilent SB-C18 (1.8 μm, 2.1 mm × 100 mm); mobile phase, mobile phase A: 0.1% formic acid aqueous solution; mobile phase B: 0.1% formic acid acetonitrile; elution gradient, the proportion of phase B is 5% in 0.00 min; the proportion of phase B increases linearly to 95% in 9.00 min, and maintains at 95% for 1 min; 10.00-11.10 min, the proportion of phase B decreases to 5%, and equilibrates at 5% to 14 min; flow rate, 0.35 mL/min; column temperature, 40 ℃; injection volume, 4 µL [56].

### ESI-MS/MS analysis

Linear ion trap (LIT) and triple quadrupole (QQQ) scans were obtained from a triple quadrupole-linear ion trap mass spectrometer (QTRAP) LC-MS/MS system, fit out with an ESI turbo ion-spray interface. The system was proceeding in both positive and negative ion modes and performed by Analyst 1.6.3 software (Sciex). The ESI source process parameters were set as follows: source temperature was set at 500 ℃; ion spray voltage (IS) were set at 5500 V (positive), and − 4500 V (negative); ion source gas I (GSI), gas II (GSII), curtain gas (CUR) were respectively set at 55, 60, and 25 psi; the collision gas (CAD) was high. 10 and 100 µmol/L polypropylene glycol solutions with QQQ and LIT modes were applied to instrument tuning and mass calibration. QQQ scans were obtained as multiple reaction monitoring (MRM) tests, and collision gas (nitrogen) was 5 psi. Individual MRM transitions based on declustering potential (DP) and collision energy (CE) were optimized by further DP and CE optimization. A specific group of MRM transitions were detected for each process on the basis of the metabolites eluted in this period [57].

### Metabolite identification and quantification

The metabolites were identified and quantified through mass spectrometry according to secondary spectrum information of metabolites in self-built MetWare library [58]. The isotopes signals, repeating signals of ions (K^+^, Na^+^ and NH_4_^+^) and other high molecular weight fragment ions were deleted. The characteristic ions of every metabolite were recognized through the QQQ rod, and the signal strength of ions were detected with the detector. Then, the acquired peaks were processed by utilizing the MultiQuant software (SCIEX) to evaluate metabolites. Each sample extraction mixture was mixed as quality control (QC) samples to verify the reliability and repeatability of the analysis results. The merged exhibition of total ion current (TIC) was used to ensure metabolite detection more accurate and reduplicative based on mass spectrometer in MRM mode (Fig. S2). Chromatographic peaks of the identified metabolites was integrated and calibrated by using MultiaQuant software [59]. The relative content of the relevant metabolite was calculated on the basis of the area of the mass spectral peaks.

### Data analysis

PCA and SCA were applied to estimating the repeatability of the samples within group and the QC samples. The identified metabolites are mapped to KEGG databases for analysis of classification and pathway information. The difference multiples were calculated based on the information of grouping. The significance of difference (P value) of each compound was examined based on T test. Orthogonal projections to latent structures-discriminant analysis (OPLS-DA) modeling was proceeded with the R language package ropls, and 200 times of permutation tests was running to examine the model reliability. Multiple cross-validation was used to obtain VIP value of the model. The difference multiple, together with the P value and the VIP value of the OPLS-DA model were applied to the filtration of the differential metabolites. The filtrating threshold are FC > 1, P < 0.05 and VIP ≥ 1.

### Electronic supplementary material

Below is the link to the electronic supplementary material.

Supplementary Material 1

Supplementary Material 2

Supplementary Material 3

Supplementary Material 4

## Data Availability

The metabolomics data analysed during the current study are available in the EMBL-EBI MetaboLights repository with the identifier MTBLS7978. The complete dataset is accessed here https://www.ebi.ac.uk/metabolights/MTBLS7978.

## Abbreviations

ANOVA: Analysis of variance
CE: Collision energy
DFMs: Differential flavonoid metabolites
DP: Declustering potential
FC: Fold change
Fh: Flowers
KEGG: Kyoto encyclopedia of genes and genomes
Lh: Leaves
LIT: Linear ion trap
MRM: Multiple reaction monitoring
OPLS-DA: Orthogonal projections to latent structures-discriminant analysis
PCA: Principal component analysis
QC: Quality control
QQQ: Triple quadrupole
Rh: Roots
SCA: Spearman correlation analysis
Sh: Stems
UPLC-MS/MS: Ultra performance liquid chromatography–tandem mass spectrometry
VIP: Variable importance in the projection

## References

[1] Ou X, Liu G, Wu L (2020). The complete chloroplast genome of *Hemerocallis citrina* (Asphodelaceae), an ornamental and medicinal plant. Mitochondrial DNA B Resour.

[2] Liu J, Zhong X, Jiang Y, Yu L, Huang X, Dong Z, Yang S, He W, Zeng J, Qing Z (2020). Systematic identification metabolites of *Hemerocallis citrina* Borani by high-performance liquid chromatography/quadrupole-time-of-flight mass spectrometry combined with a screening method. J Pharm Biomed Anal.

[3] Qing Z, Liu J, Yi X, Liu X, Hu G, Lao J, He W, Yang Z, Zou X, Sun M, Huang P, Zeng J (2021). The chromosome-level *Hemerocallis citrina* Borani genome provides new insights into the rutin biosynthesis and the lack of colchicine. Hortic Res.

[4] Li X, Jiang S, Cui J, Qin X, Zhang G (2022). Progress of genus *Hemerocallis* in traditional uses, phytochemistry, and pharmacology. J Hortic Sci Biotechnol.

[5] Han Z, Zhang H, Zhang K, Zhang X (2020). Analysis of developmental advantages of Datong daylily industry. Hortic Seed.

[6] Yang Y, Qin N, Huang J, Guo A, Xing G (2021). Dynamic changes of pectin epitopes and daylily tepals during flower opening and senescence of *Hemerocallis citrina*. Sci Hort.

[7] Xu T, Wang Y, Lu C, Feng L, Fan L, Sun J, Fan B, Wang Q, Liu X, Wang F (2020). Urinary metabolomics analysis of the anti-depressive effects of *Hemerocallis citrina* extracts in a simulated microgravity-induced rat model of depression. J Chin Pharm Sci.

[8] Zhang Y, Zhao Y, Ou Q (2020). Research progress on the post-harvest processing and medicinal mechanism of *Hemerocallis citrina* Baroni. J Anhui Agricultural Sci.

[9] Wang S, Tu H, Wan J, Chen W, Liu X, Luo J, Xu J, Zhang H (2016). Spatiotemporal distribution and natural variation of metabolites in citrus fruits. Food Chem.

[10] Dong X, Chen W, Wang W, Zhang H, Liu X, Luo J (2014). Comprehensive profiling and natural variation of flavonoids in rice. J Integr Plant Biol.

[11] Abidi J, Ammar S, Ben Brahim S, Skalicka-Woźniak K, Ghrabi-Gammar Z, Bouaziz M (2019). Use of ultra-high-performance liquid chromatography coupled with quadrupole-time-of-flight mass spectrometry system as valuable tool for an untargeted metabolomic profiling of *Rumex tunetanus* flowers and stems and contribution to the antioxidant activity. J Pharm Biomed Anal.

[12] LeVatte M, Keshteli AH, Zarei P, Wishart DS (2022). Applications of metabolomics to precision nutrition. Lifestyle Genom.

[13] Kanehisa M, Goto S (2000). KEGG: Kyoto Encyclopedia of genes and genomes. Nucleic Acids Res.

[14] Ma G, Shi X, Zou Q, Tian D, An X, Zhu K (2018). iTRAQ-based quantitative proteomic analysis reveals dynamic changes during daylily flower senescence. Planta.

[15] Taguchi K, Yamasaki K, Maesaki H, Tokuno M, Okazaki S, Moriuchi H, Takeshita K, Otagiri M, Seo H (2014). An evaluation of novel biological activity in a crude extract from *Hemerocallis fulva* L. var. Sempervirens M. Hotta. Nat Prod Res.

[16] Shiao SH, Shao BR, He YQ, Yang YQ, Yang HZ, Chang YC (1962). Studies on *Hemerocallis thunbergii* baker. Ii. Effectiveness of *Hemerocallis thunbergii* in oral treatment of experimental schistosomiasis in mice. Acta Pharm Sinica.

[17] Rodriguez-Enriquez MJ, Grant-Downton RT (2013). A new day dawning: *Hemerocallis* (daylily) as a future model organism. AoB Plants.

[18] Karak P (2019). Biological activities of flavonoids: an overview. Int J Pharm Sci Res.

[19] Han JS, Lee S, HY Kim, Lee CH (2015). MS-based metabolite profiling of aboveground and root components of *Zingiber mioga* and officinale. Molecules.

[20] Topal F, Nar M, Gocer H, Kalin P, Kocyigit UM, Alwasel SH. Antioxidant activity of taxifolin: an activity-structure relationship. 2016;31(4):674–83. 10.3109/14756366.2015.1057723 26147349

[21] Zu S, Yang L, Huang J, Ma C, Wang W, Zhao C, Zu Y (2012). Micronization of Taxifolin by supercritical antisolvent process and evaluation of radical scavenging activity. Int J Mol Sci.

[22] Li Y, Li K, Su M, Leung K, Chen Y, Zhang Y (2006). Studies on antiviral constituents in stems and leaves of *Pithecellibium clypearia*. Zhongguo Zhong Yao Za Zhi.

[23] Eldahshan OA, Azab SS (2012). Anti-inflammatory effect of Apigenin-7-neohesperidoside (Rhoifolin) in carrageenin-induced rat edema model. J Appl Pharm Sci.

[24] Liang M, Li X, Ouyang X, Xie H, Chen D (2018). Antioxidant mechanisms of Echinatin and Licochalcone A. Molecules.

[25] Cheng S, Wang H, Wu D, Gao W, Lian Y (2021). Study on antioxidant and pigmeng protection *in vitro* of quercetagetin. China Food Additives.

[26] Chung T, Chuang C, Teng Y, Hsieh M, Lai J, Chuang Y, Chen M, Yang S (2017). Tricetin suppresses human oral cancer cell migration by reducing matrix metalloproteinase-9 expression through the mitogen-activated protein kinase signaling pathway. Environ Toxicol.

[27] Ho H, Lin FC, Chen P, Chen M, Hsin C, Yang S, Lin C (2020). Tricetin suppresses migration and presenilin-1 expression of nasopharyngeal carcinoma through Akt/GSK-3β pathway. Am J Chin Med.

[28] Hsu Y, Uen Y, Chen Y, Liang H, Kuo P (2009). Tricetin, a dietary flavonoid, inhibits proliferation of human breast adenocarcinoma MCF-7 cells by blocking cell cycle progression and inducing apoptosis. J Agric Food Chem.

[29] Kim J, Lee D, Lee KW, Son JE, Seo SK, Li J, Jung SK, Heo Y, Mottamal M, Bode AM, Dong Z, Lee HJ (2011). Isorhamnetin suppresses skin cancer through direct inhibition of MEK1 and PI3-K. Cancer Prev Res.

[30] Ramachandran L, Manu KA, Shanmugam MK, Li F, Siveen KS, Vali S, Kapoor S, Abbasi T, Surana R, Smoot DT, Ashktorab H, Tan P, Ahn KS, Yap CW, Kumar AP, Sethi G (2012). Isorhamnetin inhibits proliferation and invasion and induces apoptosis through the modulation of peroxisome proliferator-activated receptor γ activation pathway in gastric cancer. J Biol Chem.

[31] Panda S, Kar A (2007). Apigenin (4’,5,7-trihydroxyflavone) regulates hyperglycaemia, thyroid dysfunction and lipid peroxidation in alloxan‐induced diabetic mice. J Pharm Pharmacol.

[32] Yin F, Giuliano AE, Van Herle AJ (1999). Signal pathways involved in apigenin inhibition of growth and induction of apoptosis of human anaplastic thyroid cancer cells (ARO). Anticancer Res.

[33] DalBó S, Jürgensen S, Horst H, Ruzza AA, Soethe DN, Santos ARS, Pizzolatti MGP (2005). Ribeiro-do-Valle RM. Antinociceptive effect of proanthocanidins from *Croton celtidifolius* bark. J Pharm Pharmacol.

[34] León-González AJ, Sharif T, Kayali A, Abbas M, Dandache I, Etienne-Selloum N, Kevers C, Pincemail J, Auger C, Chabert P. Delphinidin-3-*O*-glucoside and delphinidin-3-*O*-rutinoside mediate the redox-sensitive caspase 3-related pro-apoptotic effect of blackcurrant juice on leukaemia jurkat cells. J Funct Foods. 2015;17:847–56.

[35] Al-Madhagy SA, Mostafa NM, Youssef FS, Awad GEA, Eldahshan OA, Singab ANB (2019). Metabolic profiling of a polyphenolic-rich fraction of *Coccinia grandis* leaves using LC-ESI-MS/MS and *in vivo* validation of its antimicrobial and wound healing activities. Food Funct.

[36] Kitonde C, Dossaji SF, Lukhoba CW, Wagacha JM, Xiong Q. In vitro studies of 3-*O*-Methylquercetin against phytopathogenic fungi of major cereals. J Agricultural Sci Pract. 2019;4(4):102–12.

[37] Bouhlel I, Limem I, Skandrani I, Nefatti A, Ghedira K, Dijoux-Franca M, Leila C. Assessment of isorhamnetin 3-*O*-neohesperidoside from *Acacia salicina*: protective effects toward oxidation damage and genotoxicity induced by aflatoxin B1 and nifuroxazide. J Appl Toxicol. 2010;30(6):551–8. 10.1002/jat.1525 20809543

[38] Mayr GW, Windhorst S, Hillemeier K (2005). Antiproliferative plant and synthetic polyphenolics are specific inhibitors of vertebrate inositol-1,4,5-trisphosphate 3-kinases and inositol polyphosphate multikinase. J Biol Chem.

[39] Tasdemir D, Kaiser M, Brun R, Yardley V, Schmidt TJ, Tosun F, Rüedi P (2006). Antitrypanosomal and antileishmanial activities of flavonoids and their analogues: *in vitro*, *in vivo*, structure-activity relationship, and quantitative structure-activity relationship studies. Antimicrob Agents Chemother.

[40] Sahu NP, Achari B, Banerjee S (1998). 7,3′-Dihydroxy-4′-Methoxyflavone from seeds of *Acacia farnesiana*. Phytochemistry.

[41] Shi Q, Li L, Zhou L, Wang Y (2018). Morphological and biochemical studies of the yellow and purple–red petal pigmentation in *Paeonia delavayi*. HortScience.

[42] Ateya A, Ammar N, El-Eraky W, El-Senousy W, Amer A (2016). Antiviral, cytotoxicity, antioxidant and chemical constituents of *Adansonia digitata* grown in Egypt. Int J Pharmacogn Phytochem Res.

[43] Amarowicz R, Shahidi F (2017). Antioxidant activity of broad bean seed extract and its phenolic composition. J Funct Foods.

[44] Chuang MJ, Pandey RP, Choi JW, Sohng JK, Choi DJ, Park YI (2015). Inhibitory effects of kaempferol-3-O-rhamnoside on ovalbumin-induced lung inflammation in a mouse model of allergic asthma. Int Immunopharmacol.

[45] Diantini A, Subarnas A, Lestari K, Halimah E, Susilawati Y, Julaeha E, Achmad TH, Suradji EW, Yamazaki C, Kobayashi K, Koyama H, Abdulah R. Kaempferol-3-*O*-rhamnoside isolated from the leaves of *Schima wallichii* Korth inhibits MCF-7 breast cancer cell proliferation through activation of the caspase cascade pathway. Oncol Lett. 2012;3(5):1069–72. 10.3892/ol.2012.596 PMC3389640 22783393

[46] Curir P, Dolci M, Dolci P, Lanzotti V, Cooman LD (2010). Fungitoxic phenols from carnation (*Dianthus caryophyllus*) effective against *Fusarium oxysporum* f. sp. dianthi. Phytochem Anal.

[47] Zhang X, Zeng F, Sun Z, Yang Z, Xiong Y, Xu C, Liu X, Lin J, Mu G, Xu S, Liu W (2015). Procyanidin B2 protects LPS-induced myocardial cell apoptosis. Chin Pharmacol Bull.

[48] Deng ZJ, Zhao JF, Huang F, Sun GL, Gao W, Lu L, Xiao DQ (2020). Protective effect of procyanidin B2 on acute liver injury induced by aflatoxin B1 in rats. Biomed Environ Sci.

[49] Xing J, Li R, Li N, Zhang J, Li Y, Gong P, Gao D, Liu H, Zhang Y (2015). Anti-inflammatory effect of procyanidin B1 on LPS-treated THP1 cells via interaction with the TLR4-MD-2 heterodimer and p38 MAPK and NF-κB signaling. Mol Cell Biochem.

[50] Gao W, Yu T, Li G, Shu W, Jin Y, Zhang M, Yu X (2021). Antioxidant activity and anti-apoptotic effect of the small molecule procyanidin B1 in early mouse embryonic development produced by somatic cell nuclear transfer. Molecules.

[51] Shyaula SL, Abbas G, Siddiqui H, Sattar SA, Choudhary MI, Basha FZ (2012). Synthesis and antiglycation activity of kaempferol-3-O-rutinoside (nicotiflorin). Med Chem.

[52] Shahlehi S, Azizi A, Tengah A, Amdani SN, Petalcorin MIR. Anti-hypertensive vasodilatory action of *Gynura procumbens* mediated by kaempferol 3-*O*-rutinoside. F1000Research. 2020;9:1226.

[53] Kim CH, Abstract (2017). The inhibitory role of Kaempferol-3-O-rutinoside induced AMPK activation on the growth of human breast cancer cell lines. Cancer Res.

[54] Chen W, Balan P, Popovich DG (2020). Comparison of ginsenoside components of various tissues of New Zealand forest-grown asian ginseng (*Panax ginseng*) and american ginseng (*Panax quinquefolium* L). Biomolecules.

[55] Li M, Geng W, Wang Z, Wang Q, Pang L, Wang B, Wang P, Qu F, Zhang X (2023). Analysis of the utilization value of different tissues of Taxus×Media based on metabolomics and antioxidant activity. BMC Plant Biol.

[56] Wang J, Zhang T, Shen X, Liu J, Zhao D, Sun Y, Wang L, Liu Y, Gong X, Liu Y, Zhu Z, Xue F (2016). Serum metabolomics for early diagnosis of esophageal squamous cell carcinoma by UHPLC-QTOF/MS. Metabolomics.

[57] Li W, Wen L, Chen Z, Zhang Z, Pang X, Deng Z, Liu T, Guo Y (2021). Study on metabolic variation in whole grains of four proso millet varieties reveals metabolites important for antioxidant properties and quality traits. Food Chem.

[58] Chen W, Gong L, Guo Z, Wang W, Zhang H, Liu X, Yu S, Xiong L, Luo J (2013). A novel integrated method for large-scale detection, identification, and quantification of widely targeted metabolites: application in the study of rice metabolomics. Mol Plant.

[59] Fraga CG, Clowers BH, Moore RJ, Zink EM (2010). Signature-discovery approach for sample matching of a nerve-agent precursor using liquid chromatography-mass spectrometry, XCMS, and chemometrics. Anal Chem.

