# Supplementary material for: Comparative analysis of flavonoid metabolites from different parts of Hemerocallis citrina
Source: BMC Plant Biol. 2023 Oct 13;23:491. doi: 10.1186/s12870-023-04510-6 (PMC10571393; doi:10.1186/s12870-023-04510-6)
Supplement: Supplementary file 3 — Supplementary Material 3 [file 12870_2023_4510_MOESM3_ESM.docx]

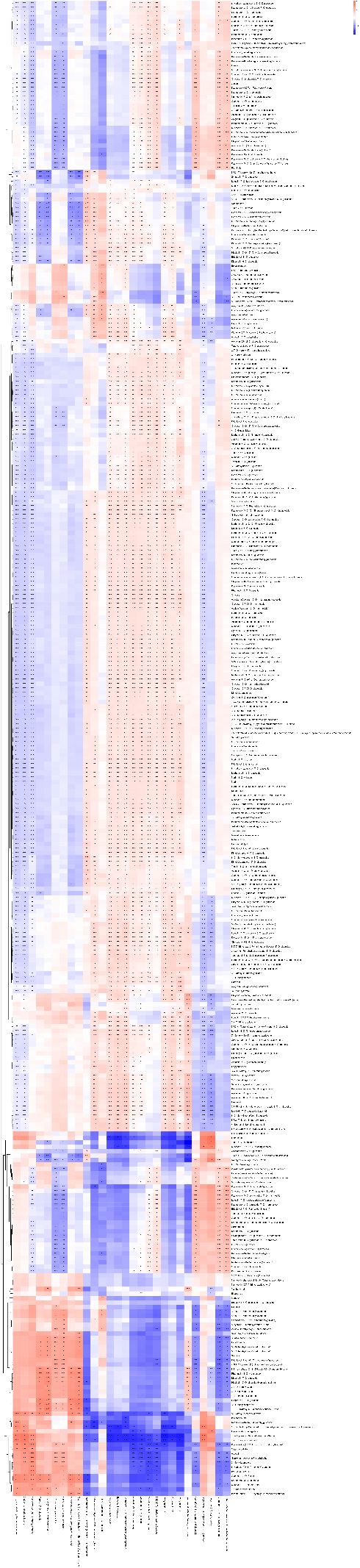


Figure S1. Correlation analysis between the dominant flavonoid metabolites in roots, stems, leaves, flowers and other detected flavonoid metabolites. * represent significant correlations at P < 0.05. ** represent significant correlations at P < 0.01. *** represent significant correlations at P < 0.001. Shades of red represent various degrees of positive correlations and shades of blue indicate various degrees of negative correlations as indicated in the scale bar on the right of the heat map.
