# Supplementary figures and images for: Comparative analysis of flavonoid metabolites from different parts of Hemerocallis citrina
Source: BMC Plant Biol. 2023 Oct 13;23:491. doi: 10.1186/s12870-023-04510-6 (PMC10571393; doi:10.1186/s12870-023-04510-6)

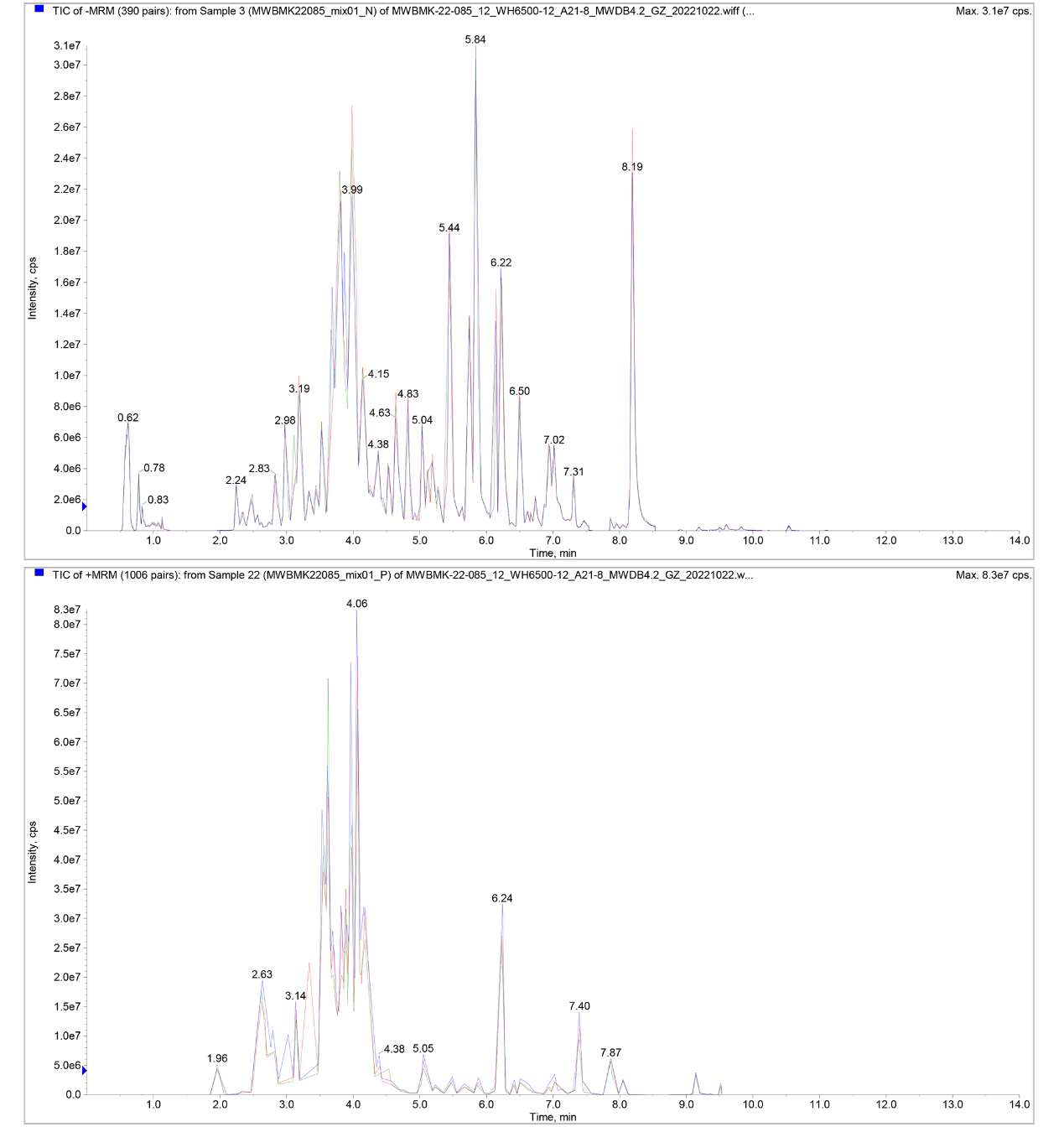


Figure S2. Detection of the TIC overlap map by QC sample mass spectrometry.

Supplement: Supplementary file 4 — Supplementary Material 4 [file 12870_2023_4510_MOESM4_ESM.docx]
